# Thermally Engineered Nickel-Tungsten Oxide Films for Energy Efficient Electrochromic Devices

**DOI:** 10.3390/nano16060375

**Published:** 2026-03-20

**Authors:** Usha K.S., Sang Yeol Lee

**Affiliations:** 1 Department of Semiconductor and Electronic Engineering, Gachon University, Seongnam-si 13120, Republic of Korea; ushakrishna1@gachon.ac.kr; 2 Gachon Advanced Institute of Semiconductor Technology, Gachon University, Seongnam-si 13120, Republic of Korea

**Keywords:** nickel tungsten oxide, electrochromic devices, smart window, sputtering, energy storage device

## Abstract

Nickel-oxide-based anodic electrochromic materials are extensively utilized as counter electrodes in smart window systems due to their reversible optical response during ion insertion and extraction. This study systematically investigates the influence of substrate temperature on the electrochromic properties of sputtered nickel-tungsten oxide thin films. The deposited thin films exhibit an amorphous structure. An increase in substrate temperature results in a decrease in nickel-vacancy concentration. Raman spectroscopy verifies the amorphous nature. Films deposited at lower substrate temperatures exhibit superior electrochromic performance, characterized by improved optical contrast of 64% and rapid coloration (2.21 s) and bleaching (0.93 s) dynamics. The enhanced performance is ascribed to the disordered amorphous structure and the existence of enough nickel vacancies, which collectively facilitate efficient and reversible lithium-ion transfer. This study illustrates that meticulous regulation of substrate temperature is an effective method for adjusting the microstructure and defect chemistry of nickel–tungsten oxide thin films, rendering them appropriate as effective counter electrodes for energy-efficient smart window applications.

## 1. Introduction

The advancement of production has correlated with an increasing need for energy consumption, leading to a succession of energy crises and environmental pollution issues. Low carbon, saving energy, and lowering emissions have become very important global problems. Nearly 40% of all greenhouse gas emissions come from buildings, and due to their poor insulation capacity, glass doors and windows account for 30–50% of building energy use [1,2,3]. A typical electrochromic device (ECD) contains a transparent conductor, electrochromic film, electrolyte layer, and ion storage film [4,5]. Metal coordination complexes (e.g., polymeric, evaporated, sublimed films), conjugated conducting polymers, viologens (as polymeric films), and transition metal oxides are common EC materials that have been researched [6,7]. Thin-film transition metal oxide electrochromism is popular due to its composition/structure diversity and outstanding electrochromic performance. Electrochromic layers, made mostly of WO_3_ and other transition metal oxides like NiO, can change their optical properties—typically their transmittance in visible light—and easily return to their bleached original condition when subjected to a voltage pulse. Tungsten oxide (WO_3_) is a good choice for electrochromic applications due to its excellent electronic conductivity (ranging from 10^6^ to 10 S cm^−1^), environmental sustainability, remarkable electrochemical stability, affordability, and diverse crystalline phases and oxidation states (W^2+^–W^6+^) [8,9]. As a result of its cost-effectiveness, compatibility, and simplicity of preparation, NiO has been widely used in commercial EC smart windows so far. Nonetheless, the disadvantages, including poor conductivity, limited optical modulation, and reduced cycle life, have emerged as substantial impediments.

Hence, recent significant advancements in transition metal oxide-based electrochromism include the development of novel materials [10], the introduction of innovative nanostructures [11,12,13], element doping [14,15], and the creation of composites [14,16,17,18]. In this scenario, Ni-W oxide composites, either tungsten incorporated into NiO [19,20] or nickel incorporated into tungsten oxide [21], demonstrate exceptional electrochromic performance [22,23]. Optimizing the thin-film deposition parameters is key to ECD efficiency. Critical factors include deposition temperature, oxygen relative pressure, and substrate temperatures affect film structural, optical, and electrical properties. Thermally treated film can modify the structure, improving ECD efficiency [24]. In a work by Xi Chen, thin films heated to 200◦C showed improved electrochromic performance, including higher optical modulation and cycle stability [25].

The current research aims to enhance the electrochromic performance of the device through tungsten incorporation into the NiO matrix and thermal treatment. Nickel-tungsten oxide thin films (NWT) were fabricated through RF sputtering at varying substrate temperatures (ST), such as room temperature (RT), 150 °C, 250 °C, 350 °C, and 450 °C. The research suggests that the low-temperature heat treatment at 150 °C developed Ni vacancies, which build multiple diffusion routes that permit ions to travel quickly. Electrochromic properties of the NWT2 material included 64% visible transmittance modulation and a hasty-switching kinetics of 0.93 s in transparent condition and 2.21 s for opaque (colored) states. These results demonstrate that controlled substrate temperature is an effective strategy for improving the electrochromic performance of NWT films.

## 2. Experimental Details

Nickel-tungsten oxide thin films, designated as NWT films, have been coated on FTO substrates using the RF-magnetron sputtering technique. The cathode made from the NWT (82%:18%) target has been placed inside the chamber for deposition. A chamber pressure of 5 × 10^−4^ mbar is soon achieved using the vacuum equipment. Argon (Ar), a noble gas, is introduced into the chamber to increase the deposition rate. The inside pressure is increased to 5 × 10^−2^ mbar and maintained at this setting over the deposition procedure. The NWT thin films were deposited as a thin layer over FTO substrates. The deposition takes place at substrate temperatures: room temperature (RT), 150 °C, 250 °C, and 350 °C, for a duration of 30 min, employing 100 W RF power.

The crystal structure was analyzed using a RIGAKU Smartlab X-ray Diffractometer (Rigaku Corporation, Tokyo, Japan). Raman spectra were recorded using STR Raman spectroscopy (Renishaw plc, Gloucestershire, UK). The chemical states of the samples were confirmed using X-ray photoelectron spectroscopy (XPS: JEOL PS-9010 TR, JEOL Ltd., Tokyo, Japan). Photoluminescence (PL) measurements were carried out using a Varian Cary Eclipse fluorescence spectrophotometer (Agilent Technologies, Santa Clara, CA, USA). The electrochemical measurements, including cyclic voltammetry (CV), chronoamperometry (CA), and chronocoulometry (CC), were performed using a scanning potentiostat/galvanostat (CHI600E, CH Instruments Inc., Austin, TX, USA). Optical measurements were performed using a UV–Vis–NIR spectrophotometer (Carry 5000 UV-Vis-NIR, Agilent Technologies, Santa Clara, CA, USA). XPS data were analyzed using CasaXPS software (version 2.3.24).

## 3. Results and Discussion

### 3.1. Structural Analysis

**Figure 1:**
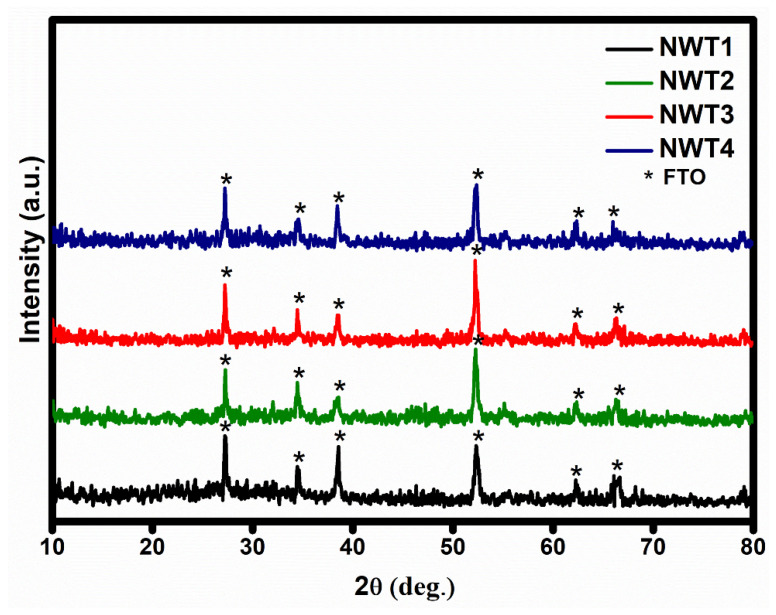
XRD patterns for NWT1, NWT2, NWT3, and NWT4 thin films.

The thickness of the NWT1, NWT2, NWT3, and NWT4 thin films was measured using a surface profilometer (Dektak XT, KLA Corporation, Milpitas, CA, USA) and found to be 0.85, 0.90, 0.97, and 1.05 μm. Figure 1 illustrates the X-ray diffraction patterns of the deposited NWT films. Only peaks corresponding to FTO substrates were observed. The absence of peaks corresponding to pristine NiO or WO_3_, or nickel-tungsten oxide composite films, confirms the amorphous nature of the deposited films. Our previous research based on pure NiO proves the crystalline nature of the deposited films [26]. Hence, the observed amorphous nature is due to the inclusion of tungsten oxide into the nickel oxide structure. Inclusion of tungsten oxide disrupts the long-range order of NiO structures due to the massive difference between the valence states of tungsten (6+) and nickel (2+). Similar results were reported for nickel-tungsten oxide composite films [27]. The films remained amorphous even with increasing the substrate temperature up to 350 °C. The observed highly disordered amorphous state of the films, irrespective of deposition temperature, paves the way for the insertion of ions, hence enhancing the electrochromic efficiency.

### 3.2. Raman Analysis

**Figure 2:**
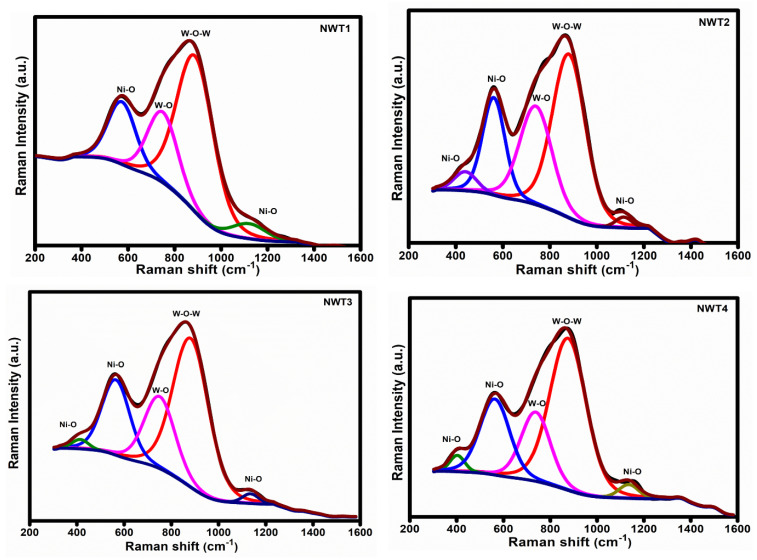
Raman spectra of NWT1, NWT2, NWT3, and NWT4 thin films. The black lines represent the experimentally measured Raman spectra, while the red, blue, green, and magenta lines correspond to the deconvoluted peaks associated with W–O–W, Ni–O, Ni–O (low-frequency mode), and W–O vibrational modes, respectively. The dark blue line indicates the baseline used for peak fitting.

The vibrational analysis for the coated NWT1, NWT2, NWT3, and NWT4 thin films is shown in Figure 2. The Raman spectra of all the samples are broader in nature, indicating the amorphous property. Previous research reported similar amorphous properties for mixed and doped electrochromic metal oxides even at higher temperatures [28,29]. These results are in line with our XRD reports. The observed broader band between 500 and 700 cm^−1^ belongs to Ni-O vibrations, while the broader band observed at 750–950 cm^−1^ represents stretching modes of W-O [30,31]. With increasing substrate temperature, there is only a small variation in the Raman intensity. Still exhibiting broader Raman bands even at higher substrate temperature, indicating the amorphous structure. The Raman analysis demonstrates the formation of highly disordered Ni-O-W structures, which supports ion mobility [29].

### 3.3. Photoluminescence Analysis

**Figure 3:**
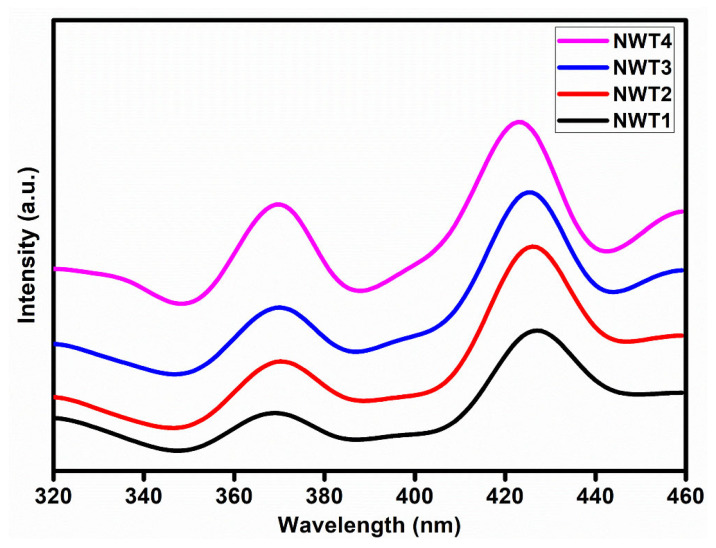
Photoluminescence spectra for NWT1, NWT2, NWT3, and NWT4 thin films.

Figure 3 shows the photoluminescence spectra for NWT1, NWT2, NWT3, and NWT4 samples. Photoluminescence studies were carried out at an excitation wavelength of 270 nm. The film showed broader emission peaks between 330 and 450 nm. This indicates the presence of near-band edge emission as well as the defect-related emissions. A weak emission band is observed near 370 nm, which originated due to the excitonic transition. This broader emission peak intensity increased with thermal treatment because of enhanced short-range ordering and the recombination process [32]. This is consistent with the Raman results. A broader blue emission is observed around 420 nm, which is associated with intrinsic defects [33]. However, the enhanced intensity with substrate temperature is due to the reduced non-radiative recombination process and increased short-range ordering of the lattice.

### 3.4. XPS Analysis

**Figure 4:**
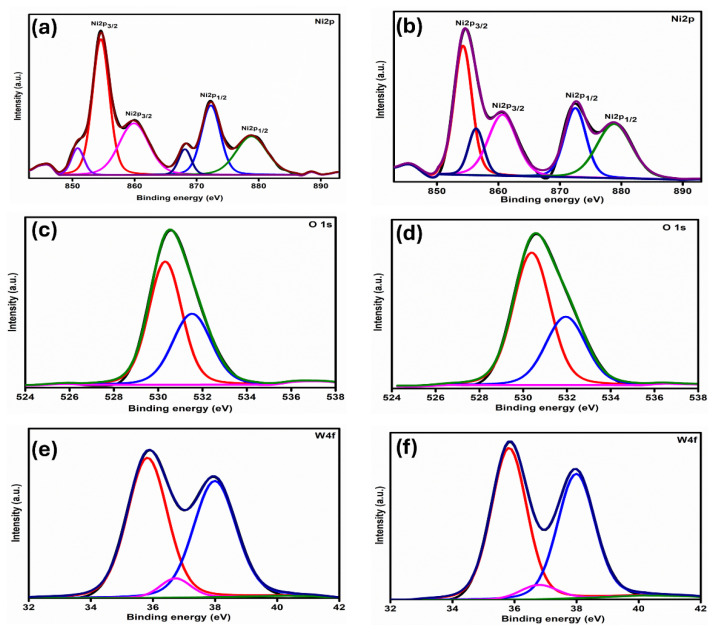
XPS core-level spectra of Ni 2p, O 1s, and W 4f for NWT2 and NWT4 thin films: (**a**,**b**) Ni 2p spectra, (**c**,**d**) O 1s spectra, and (**e**,**f**) W 4f spectra. The black lines represent the experimentally measured spectra, while the colored curves correspond to the fitted components. In the Ni 2p spectra, the red and blue peaks represent Ni²^+^ and Ni³^+^ states, respectively, and the green peaks correspond to satellite features. In the O 1s spectra, the red, blue, and green peaks are attributed to lattice oxygen, defect-related oxygen, and surface-adsorbed oxygen species, respectively. In the W 4f spectra, the red and blue peaks correspond to W^6+^ doublet components (W 4f_7/2_ and W 4f_5/2_), while the magenta curve represents the background.

Figure 4a,b shows the high-resolution Ni 2p spectra for NWT2 and NWT4 films, displaying peaks with the binding energies of 850.53 and 854.57, indicative of Ni 2p_3/2_, alongside their respective satellites around 859.90. Further peaks seen at 868.10, 872.29 correspond to the Ni 2p_1/2_ level, accompanied by their satellite at 878.84. The peaks spotted at 854.57, 872.29 eV signify the existence of Ni^3+^ states [30]. As shown in the figure, in comparison to the NWT2 film, the NWT4 film exhibits a reduced number of Ni^3+^ ions. Comparable findings were given in L. Ai et al.’s research findings [34]. Figure 4c,d represents the core level spectra for the O 1s spectra for NWT2 and NWT4 films. The peaks seen at 529.89 eV signify Ni-O (or) lattice oxygen. The peak observed at 531.45 eV corresponds to the Ni^3+^ state or to nickel vacancies and oxygen defects present on the film’s surface. The concentration of Ni^3+^ or nickel vacancies diminished with rising ST. Figure 4e,f depicts the core level spectra of W-4f for NWT2 and NWT4 thin films, revealing three distinct binding energy peaks at 35.82 eV (W 4f_7/2_), 36.78 eV (W 4f_7/2_), and 37.90 eV (W 4f_5/2_). The measured binding energies for tungsten suggest the existence of tungsten in its hexavalent form (W^6+^). The XPS examination reveals that the synthesized films are non-stoichiometric, comprising Ni^3+^ and Ni^2+^ ions. As shown in Figure 4 and Table 1, the Ni 2p data indicate that NWT2 comprises 70.02% Ni^2+^ and 29.98% Ni^3+^, while NWT4 consists of 84.16% Ni^2+^ and 15.84% Ni^3+^, suggesting a reduction in nickel-vacancy-related defects with increasing substrate temperature. O 1s analysis indicates that NWT2 contains 59.83% lattice oxygen and 40.17% defect oxygen, whereas NWT4 exhibits 64.27% lattice oxygen and 35.73% defect oxygen, implying a slight decrease in oxygen-vacancy-related defects at increased substrate temperatures.

### 3.5. Electrochromic Studies

**Figure 5:**
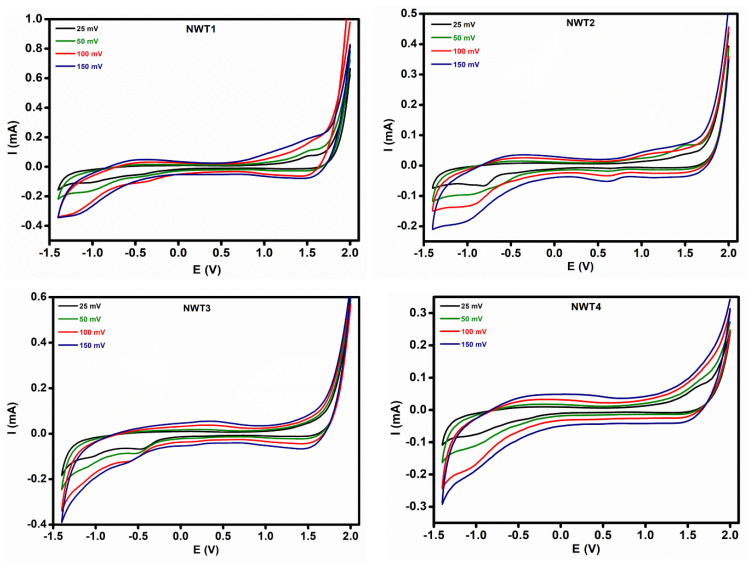
Cyclic voltammetry studies for NWT1, NWT2, NWT3, and NWT4 thin films.

The electrochromic effect associated with the ECD is analyzed via CV measurements. The CV observations for NWT1, NWT2, NWT3, and NWT4 films are presented in Figure 5, with scan rates of 25, 50, 100, and 150 mVs^−1^. Li^+^ ions are intercalated and deintercalated utilizing an electrolyte composed of 1 M LiClO_4_ distributed in propylene carbonate, across a potential scan ranging from −1.4 V to +2.0 V. NWT films undergo bleaching and coloration via the insertion and extraction of Li^+^ ions. Their response mechanism is outlined as follows.

$${NiO}_{x}+y{Li}^{+}+ye^{-}\to {Li}_{y}{NiO}_{x}$$

$$\begin{array}{c} {Li}_{y}{NiO}_{x}\;\;\;\;\;\;\;\;\;\;↔\;{Li}_{y-z}{NiO}_{x}+z{Li}^{+}+ze^{-}\\ \text{Colored (brownish) Bleached} \end{array}$$

During cathodic probing at 2 and −1.4 volts, electrons plus Li^+^ ions are transferred into the films. The insertion of Li^+^ ions generates discrete energy levels near the valence band. Discharged electrons occupy energy states in the NWT film. Additional electrons occupy energy levels in a sequence, raising the Fermi levels. This process converts Ni^3+^ to Ni^2+^ and makes the film transparent. The ECD constructed from the NWT sample permits the transmission of both visible and NIR radiation. Figure 5 illustrates that the anodic voltage scan ranging from 1.4 to −2 V facilitates the de-intercalation of Li^+^ ions, resulting in a downward shift towards the Fermi level into low-energy states and the oxidation of Ni^2+^ into Ni^3+^. This process induces a brown coloration, partially permitting visible and near-infrared (NIR) radiations.

The electrochromic characteristics are significantly influenced by the diffusion of ions through the electrode. To analyze the kinetics of the Li^+^ ion process, the diffusion coefficient was evaluated using the following relation:

$$D^{\frac{1}{2}}\;=\;\frac{i_{p}}{(2.69\times 10^{5})\times C_{o}n^{\frac{3}{2\;}}\upsilon^{\frac{1}{2}}}$$

The variables of the equation are: D represents the degree of diffusion, C_0_ denotes the quantity of electrolyte cations, n signifies the number of participating electrons, and υ indicates the scan rate. The diffusion coefficients presented in this study were estimated using the anodic peak current (*ip*) extracted from the oxidation peak in the potential range of approximately 0.9–1.5 V obtained from the CV curves. To achieve an enhanced diffusion rate, it is essential to ensure a clear and unobstructed pathway for Li^+^ ions to traverse the films in both directions. Table 1 indicates that the diffusion coefficient for the NWT2 film is greater than that of the NWT4 film. An increase in substrate temperature resulted in diminished diffusion coefficients. The transport of ions is significantly affected by the substrate temperature. This decrease in diffusion coefficient may be due to the obstruction of ion intercalation at higher temperatures [35]. Despite the driving force from the applied voltage, the dense film structure developed at higher temperatures obstructed the intercalation of Li^+^ ions, hence reducing the diffusion coefficient [36]. NWT2 film deposited at a lower temperature maintained its highly disordered structure, potentially providing numerous diffusion channels. This would facilitate the rapid movement of electrolyte ions, thus accommodating an extensive number of ions [37]. Furthermore, the XPS results indicate that the existence of Ni defects or Ni^3+^ ions may alter this diffusion pathway, hence influencing the diffusion capacity [25]. The enhanced Ni vacancies, together with the advantageous amorphous structure of the fabricated NWT2 films, create a distinctive path for Li^+^ ions, hence improving the electrochromic performance of the device.

**Figure 6:**
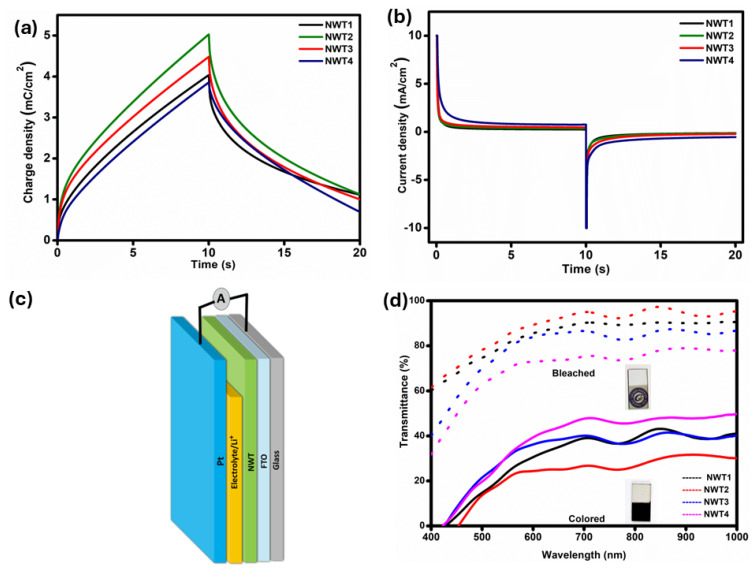
(**a**) Chronocoulometry curves of NWT1, NWT2, NWT3, and NWT4 thin films. (**b**) Time-dependent current density response of NWT1, NWT2, NWT3, and NWT4 thin films. (**c**) Schematic illustration of the NWT-based electrochromic device structure. (**d**) Transmittance spectra in colored and bleached states for NWT1, NWT2, NWT3, and NWT4 thin films. The black, red, blue, and magenta solid lines correspond to NWT1, NWT2, NWT3, and NWT4 samples, respectively (inset shows digital images of bleached and colored states for the NWT2 electrode).

Chronocoulometry (CC) gives quantitative measurements of the amount of protons as well as ions incorporated or released when a double-step potential is applied over a specified duration. Figure 6a shows the CC curves for NWT2 to NWT4 films, in which the quantity of charge intercalation (Q_in_) increased with the application of 150 °C. But further increasing the substrate temperature diminished the Q_in_ values. This may be due to the highly disordered amorphous nature of the NWT2 film.

Figure 6b illustrates the chronoamperometry curve obtained in 1 M LiClO_4_ during a duration of 10 s, with voltage ranges from +2 V to −1.4 V for Li^+^ ions. Table 2 presents the switching times for bleached (T_b_) and colored (T_c_) states. The NWT2 thin film exhibited reduced T_b_ and T_c_ values. This drop in the rate of switching is attributed to the improvement in electron conductivity and the decrease in diffusion length [38].

The main performance metrics of electrochromic materials encompass the transmittance in the bleached state (T_b_) and the colored state (T_c_). An optimal electrochromic film must demonstrate increased transparency in its bleached state and significant light absorption in its colored state. The differences between these states define the optical modulation, represented as ΔT = T_b_ − T_c_. Figure 6c shows the electrochromic device structure with NWT film as the counter electrode. Figure 6d presents the transmittance spectra for NWT samples recorded in both the colored and bleached conditions across the wavelength range of 300–1000 nm. As shown in Figure 6d, NWT1 shows 57% of optical modulation. Increasing the substrate temperature to 150 °C increased the visible optical modulation to 64%. It is observed that a further increase in temperature reduced both the colored and bleached transmittance. These optical modulation variations are associated with the ion intercalation process. At lower substrate temperatures, the NWT films exhibit a highly disordered amorphous structure that provides multiple diffusion pathways for Li^+^ ion transport. Increasing the substrate temperature to 150 °C reduces the activation energy for ion transport and provides an ideal concentration of defects, thus accelerating Li^+^ diffusion and improving optical modulation. Further increments in temperature made the film denser and more compact. This structural densification constrains Li^+^ ion diffusion pathways, impeding ion intercalation and de-intercalation mechanisms. Thus, limiting the electrochromic efficiency. The enhanced electrochromic performance of the NWT2 film is associated with its enhanced defect density. Nickel vacancies, shown by the increased Ni^3+^ content, facilitate p-type electrical conduction via Ni^2+^/Ni^3+^ hopping. Simultaneously, oxygen-vacancy-related defects provide spatially less confined areas within the amorphous oxide matrix that promote Li^+^ migration. The presence of nickel and oxygen vacancies facilitates Li^+^ insertion and extraction, hence enhancing the diffusion coefficient and accelerating the switching kinetics of the NWT2 film.

**Figure 7:**
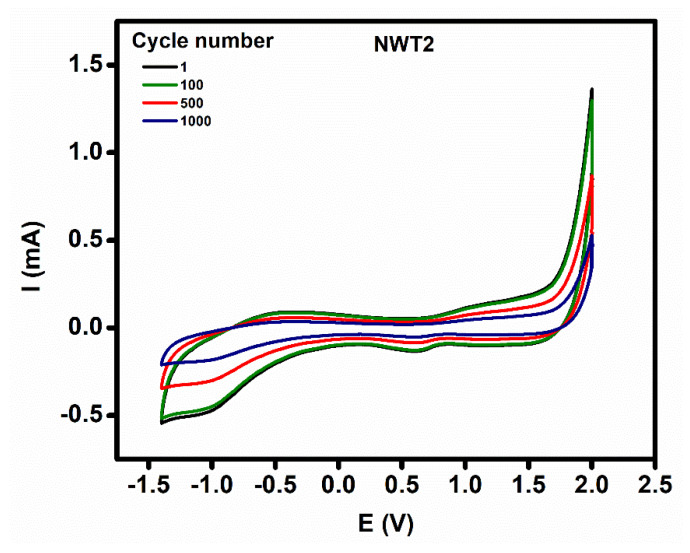
Cyclic stability of NWT2 thin films.

As shown in Figure 7, the cyclic stability of NWT2 has been assessed, indicating stability for over 100 cycles, followed by minor degradation after 500 cycles, followed by additional degradation after 1000 cycles.

## 4. Conclusions

Counter electrodes consisting of nickel–tungsten oxide (NWT) films were fabricated using RF sputtering at several substrate temperatures: room temperature (RT), 150 °C, 250 °C, and 350 °C. The deposited films were amorphous. Raman analysis verified the existence of an amorphous structure. The XPS measurements indicated that the NW films comprise a combination of Ni^2+^ and Ni^3+^ states. The substrate temperature significantly affects the electrochromic characteristics of NiO-based films by influencing their structure as well as defect concentration. NWT2 sample fabricated at 150 °C exhibited the superior electrochromic performance with an optical modulation of 64%, coloration and bleaching time of 2.21 and 0.93 s. These results indicate that the prepared nickel-tungsten oxide film is a promising counter electrode for energy-efficient smart window applications.

## Figures and Tables

**Table 1:** Summary of XPS analysis.

Sample	Ni^2+^ (%)	Ni^3+^ (%)	Ni^2+^/Ni^3+^ Ratio	Lattice Oxygen (%)	Defect Oxygen (%)
NWT2	70.02	29.98	2.34	59.83	40.17
NWT4	84.16	15.84	5.31	64.27	35.73

**Table 2:** Electrochromic properties of NWT thin films.

Sample	Diffusion Coefficient (cm^2^s^−1^) ×10^−13^	Optical Modulation ΔT = (T_b_ − T_c_) @ 550 nm	T_b_ (s)	T_c_ (s)
NWT1	6.0	57	1.12	2.40
NWT2	21.0	64	0.93	2.21
NWT3	4.0	48	1.44	2.64
NWT4	0.9	45	1.64	2.84

## Data Availability

The data presented in this study are available on request from the corresponding author. The data are not publicly available due to privacy and ethical restrictions.
